# Identification, and treatment of disease variables (DV) with individually tailored pharmacological interventions should be a pre-requisite step prior to labelling older adults with a diagnosis of treatment resistant depression (TRD).

**DOI:** 10.1192/j.eurpsy.2026.11576

**Published:** 2026-07-31

**Authors:** A. S. S. Luthra

**Affiliations:** Program for Older Adults, Homewood Health Center, Guelph, Canada

## Abstract

**Introduction:**

Preliminary results of the identification, and treatment of DV (*index episode specifier, longitudinal course specifier and diurnal variation)* in established TRD in older adults showed 95% responded to this approach. These results justify the establishment of *apriori hypothesis* that this approach must be exhausted prior to labelling older adult patients as TRD. The results of a much larger sample size are presented to affirm this hypothesis.

**Objectives:**

I hypothesize that identification, and treatment of the DV is the key to increasing the probability of response in established TRD in older adults. The principles governing the project design were derived from the *observational case study* design under the evaluative framework of the *Continuous Quality Improvement* governance structure. Results of CQI project on a Program for Older Adults (POA), a tertiary mood disorders program at Homewood Health, Guelph, Ontario, are presented to support this *a priori hypothesis.*

**Methods:**

The principles governing the project design were derived from the *observational case study* design under the evaluative framework of the *Continuous Quality Improvement* governance structure. Results of CQI project on a Program for Older Adults, a tertiary mood disorders program at Homewood Health, Guelph, Ontario, were included in this project. Each patient was evaluated using Geriatric Depression Scale (GDS)] and Becks Depressions Inventory (BDI), on admission and at discharge. T-tests was used to establish statistical significance. The approach to identify DV was presented in an earlier abstract. Pharmacology was tailored to the identified DV in each patient while they engaged in evidence informed psychotherapies.

**Results:**

a random sample of 181 patients were evaluated. Average BDI scores on admission were **28.13** and average discharge scores were **8.59** (p<0.0000000000000000000000000000000000000000000000000000000311). Average GDS scores on admission were **21.06** and average discharge scores were **8.27** (p<0.00000000000000000000000000000000000000000000000000000189). Co-efficient of correlation between BDI and GDS for the same patients was 0.8279870037.

**Conclusions:**

Identification, and treatment of DV from a larger sample offers more strength of their role in older adults getting labelled TRD. It is reasonable to suggest that identification and treatment of DV needs to be included in the definitions prior to labelling older adults with TRD.

**Disclosure of Interest:**

None Declared

